# A Review of Proteomic Studies in Uterine Leiomyosarcoma: Biomarkers, Pathways, and Clinical Potential

**DOI:** 10.3390/cimb48090925

**Published:** 2026-09-10

**Authors:** Areti Kourti, Andigoni Malousi, Konstantina Psatha, Ioannis Kalogiannidis, Michalis Aivaliotis, Elisavet Georgiou

**Affiliations:** 1 Laboratory of Biological Chemistry, Faculty of Health Sciences, School of Medicine, Aristotle University of Thessaloniki, GR-54124 Thessaloniki, Greece; andigoni@auth.gr (A.M.); kpsatha@auth.gr (K.P.); aivaliotis@auth.gr (M.A.); elgeorgiou@auth.gr (E.G.); 2 Laboratory of Medical Biology—Medical Genetics, Faculty of Health Sciences, School of Medicine, Aristotle University of Thessaloniki, GR-54124 Thessaloniki, Greece; 3 Functional Proteomics and Systems Biology (FunPATh), Center for Interdisciplinary Research and Innovation (CIRI–AUTH), GR-57001 Thessaloniki, Greece; 4 3rd Department of Obstetrics and Gynecology, Hippokratio General Hospital of Thessaloniki, Faculty of Health Sciences, School of Medicine, Aristotle University of Thessaloniki, GR-54124 Thessaloniki, Greece; ikalogia@auth.gr

**Keywords:** proteomics, uterine leiomyosarcoma, biomarkers, signaling pathways, diagnosis, prognosis, chemoresistance, multi-omics

## Abstract

Uterine leiomyosarcoma (uLMS) is a rare but highly aggressive mesenchymal malignancy of smooth-muscle origin that represents a significant therapeutic challenge due to its poor prognosis and limited treatment options. Despite advances in molecular characterization, diagnosis remains difficult, and systemic therapies have shown limited success, contributing to a high recurrence rate and poor survival. Recent proteomic investigations have provided new insights into uLMS biology by exploring the global protein expression patterns that define malignant transformation and progression. Using high-resolution mass spectrometry (MS)-based techniques, quantitative proteomics, and integrated multi-omics approaches, researchers have begun to identify dysregulated proteins, signaling pathways, and post-translational modifications (PTMs) linked to tumor metabolism, extracellular matrix (ECM) remodeling, cell-cycle regulation, and chemoresistance. These studies have also uncovered candidate biomarkers that may improve the discrimination of uLMS from benign leiomyoma and have proposed novel therapeutic targets associated with metabolic reprogramming, kinase activation and tumor microenvironment (TME) modulation. This review summarizes recent advancements in uLMS proteomics, discusses their methodological underpinnings, highlights key molecular mechanisms and biomarkers, and explores their translational potential. Finally, we outline current limitations and future directions toward clinical implementation of proteomic findings in precision oncology for uLMS patients.

## 1. Introduction

### 1.1. Background on Uterine Leiomyosarcoma

Uterine leiomyosarcoma (uLMS) accounts for approximately 1–2% of all uterine malignancies but represents nearly 70% of uterine sarcomas [1]. The disease is characterized by its aggressive behavior, high recurrence rate, and poor prognosis, with 5-year survival rates remaining below 40% for patients with advanced disease [2,3].

uLMS typically arises de novo from the myometrium, rather than progressing from benign leiomyoma, although overlapping histological and radiological features often complicate differential diagnosis [4,5]. Clinically, patients may present with abnormal uterine bleeding, pelvic pain, or a rapidly enlarging uterine mass, but these symptoms are non-specific and frequently mimic benign fibroids [6].

Even after complete surgical resection, recurrence occurs in more than half of patients, often within 18 months, and metastatic spread to the lungs, liver, and peritoneum is common [7]. Standard systemic therapies, including doxorubicin-based regimens and gemcitabine/docetaxel combinations, offer limited benefit, highlighting the urgent need for new molecularly guided strategies [7,8].

### 1.2. Current Clinical Challenges

The main clinical challenges in uLMS management stem from three interrelated issues: (i) diagnostic ambiguity, as histopathological assessment alone often fails to distinguish malignant uLMS from benign leiomyoma or atypical smooth-muscle tumors [9,10]; (ii) therapeutic inefficacy, since the disease is largely resistant to conventional chemotherapies and lacks approved targeted agents [7,11]; and (iii) high recurrence and metastatic potential, which can be attributed to early dissemination and tumor heterogeneity [2,7]. Furthermore, differentiation is frequently complicated by smooth-muscle tumors of uncertain malignant potential (STUMP), a rare gray-zone category lying histologically between benign leiomyoma and leiomyosarcoma. While preoperative imaging modalities such as MRI reveal high T2 signals, restricted diffusion, and heterogeneous architecture, aiding evaluation, definitive diagnosis still relies on histological assessment. This diagnostic ambiguity is particularly critical when managing patients desiring fertility preservation via minimally invasive or conservative uterine surgeries.

Recent genomic and transcriptomic studies have identified recurrent alterations in *TP53*, *RB1*, *ATRX*, and *PTEN* [12,13], yet these findings have not yet translated into effective therapeutic targets. Moreover, many genomic alterations in uLMS are heterogenous and context dependent, underscoring the limitations of DNA- and RNA-based approaches in capturing the functional state of tumor biology [14].

### 1.3. The Need for Molecular Understanding Beyond Genomics

Histological grading and conventional genomic analyses, though valuable, are insufficient for predicting disease behavior or treatment response in uLMS [12,14]. This gap arises because proteins are the principal effectors of cellular function, and their abundance and activity are dynamically modulated by translation rates and post-translational modifications (PTMs) [15].

Proteomics, defined as the large-scale study of the proteins of a cell or tissue, provides a detailed molecular readout of tumor physiology, capturing not only differential protein expression but also signaling activity, metabolic states, and interaction networks [16]. In the context of uLMS, proteomics has emerged as a critical tool for uncovering novel biomarkers, elucidating mechanisms of drug resistance, and identifying therapeutic targets linked to key dysregulated pathways such as energy metabolism, extracellular matrix (ECM) remodeling, and apoptosis regulation [13].

### 1.4. Scope of the Review

This review synthesizes recent advances in proteomic research related to uLMS, and organizes findings into methodological, mechanistic, and translational themes. We first outline the proteomic technologies applied in uLMS studies and describe the analytical and computational frameworks that support high-confidence protein identification, quantification, and functional interpretation. We then examine emerging proteomic insights into dysregulated signaling pathways, PTMs, and TME features that contribute to uLMS biology. In parallel, we highlight biomarker discovery efforts aimed at distinguishing uLMS from benign leiomyoma and summarize recent investigations into diagnostic, prognostic, and circulating markers. The therapeutic relevance of these findings is also discussed, including the identification of candidate drug targets and proteomic signatures associated with chemotherapy resistance. Finally, we consider the current limitations of uLMS proteomics and outline future directions, particularly the integration of multi-omics datasets and the translation of protein candidates into clinically applicable assays. Overall, this review aims to underscore the expanding role of proteomics in defining the molecular landscape of uLMS, to evaluate candidate biomarkers identified through proteomics and to assess its potential to support precision diagnostics and personalized therapeutic targets.

## 2. Literature Search Strategy

A structured literature search was conducted to identify relevant studies. Electronic databases including PubMed, Web of Science, and Scopus were queried using combinations of the following keywords: “uterine leiomyosarcoma”, “proteomics”, “mass spectrometry”, “biomarker”, “phosphoproteomics”, and “glycoproteomics”.

The search was restricted to articles published in English. No strict lower date limit was imposed to allow inclusion of foundational studies; however, emphasis was placed on research published within the past two decades, reflecting advances in proteomic technologies.

Inclusion criteria were: (i) studies employing proteomic techniques (e.g., mass spectrometry-based approaches, protein arrays, or morphoproteomics), (ii) studies involving uLMS tissue, serum/plasma, or cell-line models, and (iii) studies providing data that could be specifically attributed to uLMS or clearly separated from other sarcoma subtypes.

Exclusion criteria included: (i) studies lacking proteomic data and (ii) studies in which uLMS could not be distinguished from other soft-tissue sarcomas. Note that studies for which the full text could not be obtained after reasonable efforts were excluded from the final review; however, this represents a logistical limitation of the review process rather than a predefined scientific study selection criterion.

**Figure 1:**
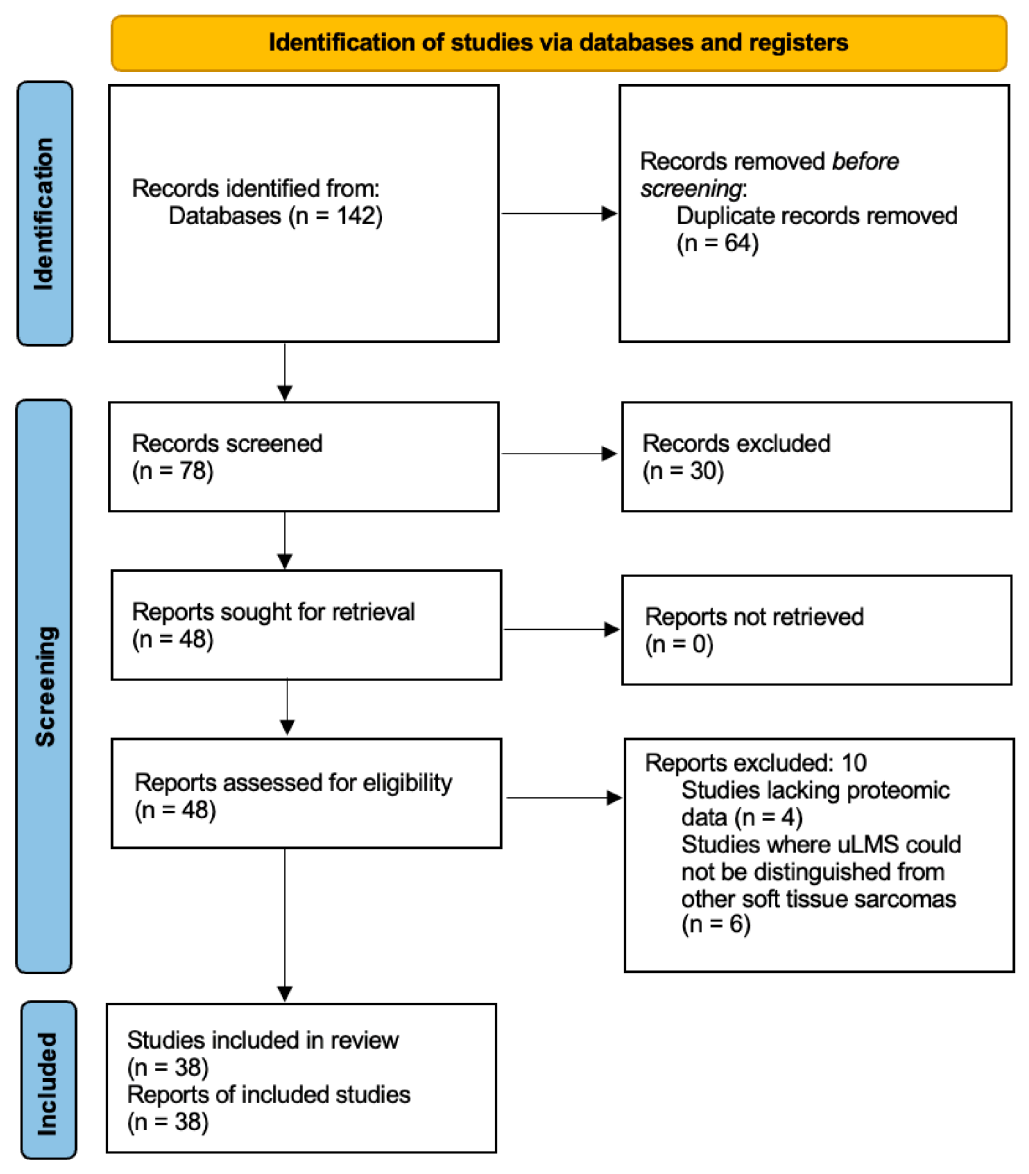
PRISMA flow diagram.

A total of 142 records were initially identified through database searching (PubMed, Web of Science, Scopus). After removing duplicates and screening titles/abstracts, 48 full-text articles were assessed for eligibility. Following application of inclusion and exclusion criteria, 38 studies were included in the final qualitative synthesis (Figure 1).

To provide clinical and biological context, selected non-proteomic studies—such as those addressing molecular diagnostics, immunohistochemical markers, and clinical management—were also included where relevant. Reference lists of included articles were manually screened to identify additional pertinent publications. The final selection reflects a curated set of studies representing the current state of proteomics research in uLMS.

## 3. Proteomic Approaches in uLMS Research

Proteomic investigation of uLMS has expanded substantially in recent years, driven by improvements in mass spectrometry (MS) sensitivity, quantitative labeling methods, and bioinformatics pipelines for data integration [12,15,16]. Given the rarity of uLMS and the challenge of obtaining well-characterized tissue specimens, proteomic studies in this malignancy have relied on carefully optimized workflows encompassing sample preparation, protein extraction, quantitative MS analysis, and computational annotation. This section outlines the major methodological domains supporting proteomic research in uLMS.

### 3.1. Data Acquisition Strategies: Data-Dependent vs. Data-Independent Acquisition

Data-dependent acquisition (DDA) has historically been the most widely used approach in uLMS proteomics. In DDA, precursor ions are selected for fragmentation based on their intensity during real-time analysis. This approach enables identification of a broad range of proteins but is inherently stochastic, leading to missing values across samples, particularly in complex clinical cohorts.

Methods using data-independent acquisition (DIA) (Table 1), an alternative that addresses some limitations of DDA, such as SWATH-MS, have emerged as a powerful approach for reproducible proteome profiling across clinical cohorts. Although not yet widely applied to uLMS, they are increasingly used in soft-tissue sarcoma proteomics. In DIA, all ions within predefined mass-to-charge windows are systematically fragmented, resulting in more consistent detection across samples. This improves reproducibility and quantitative completeness, which are critical for clinical biomarker studies. Although DDA remains prevalent in earlier uLMS studies, recent advances in instrumentation and computational analysis are driving increased adoption of DIA-based workflows, particularly in studies aiming for cohort-level comparisons [17,18].

### 3.2. Quantification Strategies: Label-Based vs. Label-Free Approaches

Quantitative proteomics can be achieved using label-based or label-free methods (Table 1).

Label-based approaches, including isobaric tagging techniques (e.g., iTRAQ) and tandem mass tag (TMT), allow multiplexing of multiple samples within a single MS run and with limited sample amounts [16]. In these methods, peptides are labeled with isobaric tags that yield specific reporter ions upon fragmentation during MS/MS. Peptide and protein quantification is therefore achieved by measuring and comparing the relative intensities of these low-mass reporter ions across the multiplexed samples, allowing for precise relative abundance calculations. This can reduce technical variability within a batch and facilitate relative quantification across conditions. However, these methods are subject to ratio compression due to co-isolation interference and often require extensive peptide fractionation to achieve deep proteome coverage, increasing experimental complexity. Ke et al. [5] utilized TMT-based proteomics to compare malignant uLMS with benign leiomyoma, identifying distinct expression patterns in ECM-related proteins and metabolic enzymes.

Label-free quantification (LFQ) methods estimate protein abundance based on peptide signal intensity or spectral counting across separate runs. LFQ estimates peptide intensity or spectral counts without constraints on the number of samples analyzed, is suitable for studies involving larger cohorts, and has gained much attention in recent studies for its flexibility and reduced cost. It is particularly advantageous in rare tumors where sample quantity is limited [13]. When combined with robust normalization and quality control, LFQ can achieve high reproducibility and quantitative accuracy.

### 3.3. Relevance to uLMS Studies

In comparative analyses, iTRAQ and TMT labeling have demonstrated robust reproducibility and sensitivity for detecting fold changes as low as 1.2–1.5 between leiomyoma and uLMS samples [19]. These studies often combine high-resolution Orbitrap mass analyzers with nano-LC separation to improve detection of low-abundance tumor-associated proteins [20]. The choice of method often reflects trade-offs between depth of coverage, reproducibility, and sample throughput. Given the rarity of uLMS and the limited availability of clinical specimens, methodological considerations play a critical role in determining the reliability and interpretability of findings. In addition to global proteomics, specialized approaches such as phosphoproteomics and glycoproteomics have been used to investigate signaling dynamics and post-translational modifications, providing additional layers of biological insight.

Two-dimensional polyacrylamide gel electrophoresis (2D-PAGE) and Western blotting represent foundational proteomic techniques that predate MS-based methods. Early investigations into uterine smooth-muscle tumors employed 2D-PAGE to identify gross differences in protein spot patterns between benign and malignant lesions [21]. Despite lower throughput and resolution, these techniques remain valuable for validation of specific targets identified by MS. For instance, Guo et al. [6] verified the differential expression of caldesmon, desmin, and tropomyosin isoforms in uLMS versus leiomyoma by Western blotting. Moreover, immunoblotting continues to serve as a confirmatory method in phosphoproteomic and glycoproteomic analyses [22].

### 3.4. Emerging Technologies

While single-cell proteomics and spatially resolved proteomics have been broadly established in pan-sarcoma cohorts, their direct application to uLMS tissue sections remains in its nascent stages, with most current high-resolution spatial maps derived from broader soft-tissue sarcoma investigations [7,8]. Laser-capture microdissection (LCM) coupled with LC-MS/MS allows selective analysis of tumor and stromal compartments, providing insight into microenvironmental interactions [5]. Furthermore, imaging MS (IMS) and matrix-assisted laser desorption/ionization (MALDI) approaches have been employed in exploratory studies of uterine sarcomas to spatially localize protein signatures associated with invasion fronts and necrotic regions [6].

### 3.5. Sample Types and Preparation

#### 3.5.1. Tissue Specimens

uLMS proteomic studies utilize both fresh-frozen and formalin-fixed paraffin-embedded (FFPE) tissue samples. Fresh tissue offers superior protein preservation but is rarely available given the disease’s rarity and retrospective nature of most studies [23]. FFPE specimens, on the other hand, constitute a vast resource in pathology archives, but formalin-induced crosslinking complicates protein extraction [11]. Recent optimization of antigen retrieval buffers and surfactant-aided lysis has significantly improved the recovery of proteins from FFPE uLMS tissue, enabling compatibility with modern MS workflows [24].

#### 3.5.2. Biofluid-Based Samples

Proteomic analysis of serum, plasma, and urine offers a minimally invasive route for biomarker discovery in uLMS. Kopelman et al. [15] performed deep serum proteome profiling of uLMS and leiomyoma patients, identifying differentially abundant acute-phase proteins, ECM regulators, and metabolic enzymes. Such circulating biomarkers hold potential for preoperative differentiation of uLMS from benign tumors—a major unmet clinical need [12]. However, biofluid proteomics faces challenges due to the high dynamic range of protein concentrations and interference from abundant plasma proteins like albumin and immunoglobulins [25]. Techniques such as immunodepletion, ultrafiltration, and peptide fractionation are routinely applied to mitigate these issues [26].

#### 3.5.3. Cell-Line and Organoid Models

Established uLMS cell lines (e.g., SK-UT-1) have facilitated mechanistic proteomic investigations, particularly in studies exploring chemotherapy resistance and signal transduction [21]. More recently, patient-derived organoids and xenografts have been used to validate proteomic signatures observed in clinical samples, allowing functional assays of targetable pathways such as mTOR, PI3K/AKT, and MAPK signaling [27]. Despite their value, cell models may incompletely recapitulate tumor heterogeneity and microenvironmental influences present in vivo.

### 3.6. Technical Challenges in uLMS Samples

Like many tissue-based proteomic studies, uLMS research faces common analytical challenges, such as tissue heterogeneity and the low abundance of certain regulatory proteins. In uLMS specifically, the admixture of large necrotic areas and dense stromal elements can significantly confound protein quantification. Necrotic cells release degraded proteins and intracellular contents that mask true tumor-specific signals, while abundant stromal proteins (e.g., collagens) can suppress the ionization and detection of lower-abundance cancer biomarkers. If left unaddressed, these issues can lead to false-positive differential expressions and reduced confidence in the proteomic data. While some studies employ laser-capture microdissection (LCM) to isolate viable tumor cells, these confounding factors are often overlooked in bulk tissue analyses, highlighting a critical limitation in interpreting existing data. Furthermore, while informatics approaches and robust normalization are essential, they alone cannot fully overcome the biological inter-patient variability or the statistical limitations imposed by the small cohort sizes characteristic of this rare tumor. Multi-institutional tissue banking remains critical.

### 3.7. Computational Workflows and Data Analysis

The complexity of proteomic datasets in uLMS requires robust computational workflows for accurate protein identification, quantification, and functional interpretation. Raw mass spectrometry data are typically processed using software platforms such as MaxQuant, Proteome Discoverer, or Mascot, with strict control of false discovery rates (FDRs) to ensure high-confidence peptide assignments [17,19]. Quantitative strategies, including label-based methods such as iTRAQ and TMT, as well as label-free quantification, require normalization and statistical analysis to distinguish true biological variation from technical noise [12,20].

Beyond protein-level analysis, computational pipelines are essential for the identification of proteoforms, including post-translationally modified, splice-variant, or proteolytically cleaved forms, which often mediate functional tumor phenotypes [8,26]. PTM-specific searches, such as phosphorylation, glycosylation, and acetylation mapping, are integrated with pathway and network analyses to reveal dysregulated signaling, metabolic, and ECM pathways [25]. Enrichment analyses using comprehensive data sources such as Gene Ontology (GO), KEGG, and Reactome further contextualize proteomic findings within biological processes and molecular functions [22].

Finally, computational approaches support translational applications, enabling the prioritization of candidate biomarkers and drug targets. Network-based scoring, machine-learning algorithms, and proteoform-aware annotation pipelines help identify the most functionally relevant proteins, guiding both mechanistic studies and potential clinical translation [7,27]. Apart from enhancing interpretability, bioinformatic strategies also underscore current limitations and challenges in proteomics: incomplete proteoform coverage, variability in database annotations, and the need for standardized pipelines across studies.

## 4. Proteomic Insights into uLMS Biology

Proteomic profiling has provided critical insights into the biological processes driving uLMS. By examining differential protein expression patterns and pathway enrichment analyses, several molecular mechanisms have emerged as central to the aggressive phenotype of this tumor: metabolic reprogramming, ECM remodeling, dysregulated cell-cycle and apoptosis control, and alterations in PTMs. These findings collectively illuminate the molecular underpinnings of tumor proliferation, invasion, and therapy resistance in uLMS [6,7,12,13].

### 4.1. Cell-Cycle Dysregulation and Proliferation

One of the most consistent findings in uLMS proteomics is the dysregulation of proteins involved in cell-cycle control. Deregulation of cell-cycle checkpoints is a defining feature of uLMS, reflected by aberrant expression of cyclins, cyclin-dependent kinases (CDKs), and tumor suppressors [6,11,13]. Proteomic analyses have revealed overexpression of cyclin B1 (CCNB1), CDK1, TOP2A, and Aurora kinase A (AURKA), all of which promote uncontrolled mitosis [6,13,23]. Elevated Ki-67 and TOP2A protein levels, validated by immunohistochemistry, are associated with poor prognosis and serve as potential prognostic biomarkers [28,29].

### 4.2. Metabolic Reprogramming and Extracellular Matrix Remodeling

Alterations in metabolic pathways are another prominent feature identified through proteomics. Increased expression of enzymes involved in glycolysis, nucleotide biosynthesis and lipid metabolism suggests a shift toward anabolic processes that support rapid tumor growth.

ECM remodeling and altered cell–matrix communication play a central role in uLMS invasion and metastasis. Comparative proteomics between uLMS and benign leiomyoma consistently reveal overexpression of structural ECM components and remodeling enzymes, including collagens I, II, and VI, fibronectin, tenascin C, matrix metalloproteinases (MMP2, MMP14), and lysyl oxidase (LOX) [6,7].

Pathway-enrichment analyses in proteomic studies of soft-tissue sarcomas often report terms such as “ECM-receptor interaction” and “focal adhesion”, suggesting that adhesion proteins, including integrins and cytoskeletal adaptors, may play key roles in uLMS biology [7]. Additionally, differential expression of ECM regulators such as microfibrillar-associated protein 5 (MFAP5) and decorin has been proposed as a diagnostic aid for distinguishing uLMS from leiomyoma [6]. MFAP5, in particular, was shown to correlate with immune infiltration and aggressive histopathological features, highlighting its dual role as a matrix protein and a signaling modulator in the tumor microenvironment (TME) [6,7]. Proteomic evidence also points to the activation of the PI3K/AKT and FAK-Src signaling pathways downstream of integrin engagement, driving cytoskeletal reorganization and enhancing metastatic potential [5]. Elevated phospho-FAK (Y397) and phospho-AKT (S473) have been detected in TMT-based quantitative datasets, underscoring a functionally active invasion program [5,22].

### 4.3. mTOR Signaling and Growth Regulation

Phosphoproteomic studies of uterine sarcomas have identified extensive kinase pathway activation consistent with the tumor’s high proliferative and migratory capacity [7,24,30]. In particular, phosphorylation of ribosomal protein S6 (RPS6) and 4E-binding protein 1 (4EBP1) indicates constitutive activation of the mTORC1 complex, which supports protein synthesis and cell growth [12,15]. Elevated phosphorylation of ERK1/2 (MAPK1/3) and MEK1/2 (MAP2K1/2) further implicates sustained MAPK signaling in tumor proliferation and invasion [7,30]. These findings suggest that kinase inhibitors targeting mTOR or MEK may have therapeutic relevance in uLMS, aligning with early preclinical data demonstrating sensitivity of uLMS models to rapalogues and MEK inhibitors [5,24].

### 4.4. Apoptosis and Therapy Resistance

Proteomic analyses indicate alterations in proteins involved in apoptotic pathways, including both pro- and anti-apoptotic regulators. Loss or downregulation of p16INK4a, RB1 and TP53 pathways disrupts the G1/S checkpoint and apoptotic response [13,31]. Phosphoproteomic profiling further revealed hyperactivation of checkpoint kinase 1 (CHEK1) and cyclin-dependent kinase (CDK2), suggesting potential vulnerabilities to cell-cycle inhibitors [24,30]. Indeed, experimental treatment of uLMS-derived cells with CDK inhibitors (e.g., palbociclib) resulted in growth suppression and partial restoration of apoptotic signaling [23,24]. Collectively, these data underscore that dysregulation of cell-cycle control and apoptosis is a proteomic hallmark of uLMS, underpinning its high proliferative index and resistance to cytotoxic therapies [6,11,23,24].

### 4.5. Glycoproteomics: Cell-Surface Markers and Metastatic Potential

Aberrant glycosylation affects cell adhesion, immune recognition, and metastatic dissemination [12,26]. Targeted glycoproteomic methodologies, such as those pioneered by Abbott et al. [32] in epithelial cancers, are increasingly being adapted to identify differential N-glycosylation profiles in mesenchymal tumors like uLMS [25]. These modifications influence cell–matrix interactions and may serve as potential biomarkers for metastatic competence [26]. Moreover, increased fucosylation and sialylation of membrane proteins such as CD44 and EGFR have been correlated with enhanced migration and doxorubicin resistance in uLMS cell models [21]. Integrative glycol- and phosphoproteomic analyses thus provide a more complete picture of signaling modulation beyond abundance changes alone.

Glycoproteomic profiling by Abbott et al. [32] established foundational N-glycosylation workflows primarily in epithelial breast carcinoma models. Although direct high-throughput glycoproteomic tissue mapping in uLMS remains limited, related cell-line investigations demonstrate that aberrant glycosylation of membrane proteins like CD44 and EGFR correlates with enhanced migration and chemoresistance [21], indicating that similar pathways likely operate in uLMS.

### 4.6. Distinguishing uLMS-Specific Findings

A critical consideration in interpreting these results is the distinction between findings derived from uLMS-specific studies and those observed in broader soft-tissue sarcomas. Many pathways identified in proteomic analyses are common across multiple cancer types. Where possible, this review prioritizes evidence directly derived from uLMS samples. However, given the limited number of studies, some insights are informed by related sarcoma research and should be interpreted with caution.

### 4.7. The Role of Tumor Microenvironment

The tumor microenvironment (TME) of uLMS is increasingly recognized as a key determinant of tumor progression and therapeutic response. Proteomic analyses have begun to unravel the crosstalk between malignant cells, immune infiltrates, and stromal components [7].

#### 4.7.1. Immune Modulation and Infiltration

Ke et al. [5] and Falcão et al. [25] observed enrichment of immune-related proteins, including complement cascade components (C3, C4A, C1QB), and inflammatory mediators (S100A8, S100A9), suggesting chronic inflammation and immune evasion. Moreover, proteomic signatures consistent with macrophage infiltration and M2 polarization, and marked with increased abundance of CD163 and ARG1 have been identified [7]. These features correlate with poor clinical outcome and resistance to chemotherapy. Elevated expression of immune checkpoint proteins such as PD-L1 and IDO1 has also been detected at the proteomic and immunohistochemical levels, implying potential benefit from immune checkpoint inhibition in selected uLMS cases [33].

#### 4.7.2. Angiogenesis and Stromal Interaction

Angiogenic proteins including VEGFA, angiopoietin-2 (ANGPT2), and endoglin (ENG) are consistently increased in abundance in uLMS tissues [30]. Proteomic expression networks highlight crosstalk between tumor and endothelial cells via the vascular endothelial growth factor (VEGF) and transforming growth factor-beta (TGF-β) pathways [34]. Stromal fibroblasts contribute additional paracrine factors such as periostin (POSTN) and thrombospondin-1 (THBS1), which remodel the ECM and promote metastatic niche formation [6,8,30]. Integration of proteomic and secretomic data has further identified soluble proteins secreted by tumor-associated fibroblasts, such as MMP14, LOXL2, and SERPINE1, that correlate with invasiveness and poor prognosis [19,30].

## 5. Proteomic Findings: Biomarker Discovery and Diagnostic Potential

One of the central goals of uLMS proteomics is to identify molecular signatures that can improve diagnosis, stratify prognosis, and enable non-invasive disease monitoring, Conventional histopathological methods often fail to distinguish uLMS from benign leiomyoma, especially in early stages or in mixed lesions, resulting in delayed or inappropriate treatment [1,6,11]. Proteomic profiling offers an objective molecular framework for discriminating malignant from benign smooth-muscle tumors and for uncovering biomarkers predictive of outcome and therapeutic response [6,7,13,19]. Table 2 provides an overview of key proteomic biomarkers reported to date across tissue-based and circulating proteome studies.

### 5.1. Tissue-Based Biomarkers

Proteomic analyses of tumor tissue have identified numerous candidate proteins that differ between uLMS and benign leiomyomas or other smooth-muscle tumors. These include proteins involved in cell-cycle regulation, stress response, cytoskeletal organization, and signaling pathways.

For clarity, biomarkers can be classified into three categories: (i) diagnostic biomarkers that distinguish uLMS from benign or borderline lesions, (ii) prognostic biomarkers that predict disease outcome or progression, and (iii) characterization biomarkers that provide insight into tumor biology without direct clinical application.

Many candidate biomarkers identified through proteomics have subsequently been evaluated using immunohistochemistry (IHC). It is important to distinguish between discovery (proteomics) and validation (IHC or targeted assays), as these represent different levels of evidence.

#### 5.1.1. Diagnostic Biomarkers

Several comparative proteomic studies have revealed characteristic expression patterns that differentiate uLMS from leiomyoma. Early 2D-PAGE studies identified overexpression of vimentin, desmin, α-smooth-muscle actin, and calponin in both tumor types, confirming smooth-muscle origin, but uLMS showed unique upregulation of heat-shock proteins (HSP27, HSP70), annexin A2, and galectin-1 [9,12,13,36].

Subsequent LC-MS/MS-based quantitative proteomics using iTRAQ and TMT labeling improved sensitivity and reproducibility. Ke et al. [5] reported that MFAP5 and dipeptidyl-peptidase 6 (DPP6) were significantly overexpressed in uLMS relative to leiomyoma and associated with immune-related pathways, suggesting dual diagnostic and immunological relevance. These findings were validated by immunohistochemistry in an independent cohort, where MFAP5 achieved an AUC > 0.90 for distinguishing uLMS from leiomyoma [5].

Proteomic datasets from Guo et al. [6] and Falcão et al. [25] identified thrombospondin-2 (THBS2), POSTN, and fibronectin (FN1) as top discriminators, reflecting ECM remodeling and invasion potential in uLMS. In contrast, leiomyoma tissues displayed higher expression of tropomyosin 1 (TPM1), smooth-muscle myosin heavy chain, and metabolic enzymes such as fructose-bisphosphate aldolase A (ALDOA), consistent with a less aggressive phenotype [14].

Recent label-free proteomics using FFPE samples from multi-center cohorts confirmed the reproducibility of these markers across different tissue preservation methods [7,19]. Enrichment analysis revealed dominant pathways, including “ECM-receptor interaction”, “focal adhesion”, and “oxidative stress response”, reflecting the malignant transformation process [6,8,19].

#### 5.1.2. Evaluation of Emerging Multi-Protein Signatures

Pathway-level analyses frequently report enrichment of ECM and adhesion-related processes in proteomic studies of uterine and soft-tissue sarcomas, and several groups have proposed multi-protein diagnostic panels that principally comprise ECM and cell-adhesion proteins; however, these candidate panels remain at the discovery or early-validation stage. Further validation in larger independent cohorts is required before classification models can discriminate uLMS from leiomyoma clinically. Identifying concrete protein candidates that can bridge tissue discovery and blood-based diagnostics is a critical next step for clinical translation. Several proteins identified in Table 2 are particularly well suited for liquid biopsy panels. Because proteins such as MFAP5, DPP6, and THBS2 are heavily involved in ECM remodeling or localized to the cell surface, they are shed, secreted, or packaged into extracellular vesicles (EVs) within the tumor microenvironment (TME) and released into systemic circulation. Recent functional assays and transcriptomic cross-validations support their robust expression in uLMS compared to benign leiomyomas. Consequently, these secreted and cell-surface markers represent ideal candidates for emerging liquid biopsy evaluations, bridging the gap between localized tissue proteomics and systemic detection frameworks, such as those actively being evaluated in the 2025 DOORS-D/M clinical trials [38].

### 5.2. Prognostic Biomarkers

#### 5.2.1. Correlates of Survival and Recurrence

Beyond diagnostic use, proteomic studies have uncovered numerous proteins linked to clinical outcome. Burns et al. [7] performed deep quantitative proteomics of multiple soft-tissue sarcoma subtypes and identified distinct proteomic clusters and subtype-specific modules. Their data emphasize broader functional signatures, such as vesicle transport and immune-related modules, that may guide future studies of sarcoma biology. In clinical samples, high topoisomerase II α (TOP2A) and Aurora kinase A (AURKA) expression correlated with reduced overall survival [6,28,34].

Further, overexpression of aldehyde dehydrogenase (ALDH1A1) and carbonic anhydrase IX (CA9), which are markers of hypoxia and stem-like features, was linked with recurrence and chemoresistance [24]. Phosphoproteomic analyses revealed that hyperphosphorylation of ribosomal S6 and 4EBP1 (mTORC1 activation) predicted poor response to standard chemotherapy [24,39].

Immune-related prognostic markers have also emerged. High MFAP5 expression correlated with increased infiltration of M2-type macrophages and unfavorable prognosis [5,6]. Conversely, elevated HLA-A and β2-microglobulin levels indicated enhanced antigen presentation and better disease-free survival [7].

#### 5.2.2. Candidate Validation Studies

Validation of proteomic discoveries through immunohistochemistry (IHC), Western blotting, or enzyme-linked immunosorbent assay (ELISA) is essential for clinical translation. Sparić et al. [31] summarized that KI-67, TOP2A, and AURKA have achieved partial validation as prognostic markers across several cohorts, whereas others such as MFAP5 and DPP6 require larger studies. Targeted mass spectrometry methods, including multiple-reaction monitoring (MRM), represent an ideal approach for the quantitative validation of low-abundance candidates, though their application in uLMS remains limited to date.

### 5.3. Circulating and Biofluid Biomarkers

Non-invasive biomarkers detectable in serum or plasma are of particular interest for improving preoperative diagnosis. Proteomic studies of circulating proteins in uLMS remain limited but have identified candidate markers associated with tumor presence and progression. Studies with larger sample cohorts have begun to address limitations of earlier work, although variability in study design and analytical methods remain a challenge.

#### 5.3.1. Serum and Plasma Proteomics (Liquid Biopsy)

Liquid biopsy-based proteomics offers a minimally invasive strategy for biomarker discovery in uterine smooth-muscle tumors. Significant progress is underway in utilizing blood markers to differentiate uterine sarcomas from fibroids pre-operatively. Although initiatives such as the DOORS-D (Diagnosis) and DOORS-M (Monitoring) studies (2025) [38] are actively evaluating liquid biopsy signatures, validated quantitative diagnostic performance metrics (such as prospective large-cohort AUC, sensitivity, and specificity) for standalone circulating proteomic markers in uLMS remain largely unestablished, emphasizing a major unmet clinical need.

Because existing blood markers like CA125 or LDH lack sufficient specificity, the integration of proteomic signatures—such as extracellular vesicles (EVs) or circulating ECM-associated proteins—with emerging genomic liquid biopsy tools represents a critical frontier for recent (2024–2026) research. For liquid biopsies to succeed clinically, the concrete protein candidates discovered in tissue phases (summarized in Table 2, such as MFAP5, DPP6, and THBS2) must be systematically evaluated in plasma or serum cohorts to establish an easily measurable and highly specific blood-based diagnostic panel.

#### 5.3.2. Urine and Other Biofluids

Although urinary proteomics is an underexplored area in uLMS, the feasibility of urinary biomarker discovery is supported by peptidomic studies in other disease contexts. For instance, urinary peptides derived from collagen degradation and matrix metalloproteinase-related fragments have been identified in kidney disease and other conditions [40,41,42]. However, as of today, no published proteomic or peptidomic study has reported urinary peptide profiles specific to uLMS, highlighting a critical gap for future investigation.

### 5.4. Current Limitations in Biomarker Development

While numerous candidate biomarkers have been reported, translation to clinical practice remains limited. Challenges include sample heterogeneity, lack of standardized pre-analytical workflows, and insufficient validation across independent cohorts [1,7]. Nevertheless, the convergence of diagnostic and prognostic data around a core set of ECM-associated proteins (FN1, COL6A3, MFAP5, THBS2), cell-cycle regulators (TOP2A, AURKA), and immune modulators (S100A8/9, PD-L1) is encouraging [5,6].

Emerging MS-based clinical platforms, such as DIA and microflow LC, offer improved reproducibility and throughput, paving the way for clinical proteomics assays [11,24]. Ultimately, a combination of tissue-based and circulating protein biomarkers, integrated with imaging and genomic information, is expected to enable early, accurate, and personalized diagnosis of uLMS [4,6,19,24].

One important consideration is the relationship between proteomics and other omics approaches. Transcriptomic profiling is often more accessible and cost effective, but it does not capture post-translational regulation or protein activity. Proteomics provides complementary information that may be particularly valuable for identifying functional biomarkers and drug targets. In cases of rare tumors, such as uLMS, proteomics may offer unique advantages in identifying disease-specific protein signatures that are not apparent in the transcript level. However, practical challenges, including cost, technical complexity, and the need for specialized expertise, limit widespread clinical adoption.

Integration of proteomic data with clinical and molecular information represents a promising strategy for improving diagnostic accuracy and guiding treatment decisions. Targeted multi-omics assays and multiplexed platforms may facilitate translation by providing more standardized and scalable approaches.

## 6. Clinical Implications

Prior to evaluating therapeutic interventions, establishing robust proteomic signatures for the differential diagnosis of uLMS from benign leiomyomas remains the paramount clinical priority.

### 6.1. Identifying Novel Drug Targets

#### 6.1.1. Proteomics-Driven Target Discovery

The proteomic landscape of uLMS has illuminated several actionable pathways that may serve as therapeutic entry points. Quantitative and phosphoproteomic studies consistently implicate aberrant activation of PI3K/AKT/mTOR, MAPK/ERK, and cell-cycle kinase cascades [6,12,21,24,39]. Overexpression and hyperphosphorylation of AKT1, mTOR, RPS6, and 4EBP1 mark sustained growth signaling, positioning these kinases as candidates for pharmacologic inhibition [22,24]. Rapamycin analogues (temsirolimus, everolimus) and dual PI3K/mTOR inhibitors have shown antiproliferative effects in preclinical uLMS models, though clinical efficacy remains modest [43].

Parallel analyses reveal dysregulated cell-cycle regulators, including AURKA, AURKB, CDK1, and TOP2A [6,11,13]. Their overexpression correlates with poor prognosis and chemoresistance, supporting the rationale for selective Aurora-kinase or CDK inhibition. Preclinical studies using palbociclib (CDK4/6 inhibitor) and alisertib (AURKA inhibitor) demonstrated reduced mitotic activity and partial induction of apoptosis in uLMS cell lines.

Proteomic evidence also underscores the therapeutic potential of targeting epigenetic modifiers. Elevated levels of histone deacetylases (HDAC1/2) and EZH2 have been detected in aggressive uLMS tumors. HDAC inhibition with vorinostat or panobinostat induces differentiation and apoptosis in vitro, while EZH2 inhibitors are being explored to suppress the oncogenic chromatin state associated with TP53 and RB1 loss.

#### 6.1.2. ECM and Adhesion Pathways

Given the strong enrichment of ECM-related proteins in uLMS [6,8,19], therapeutic disruption of tumor–matrix interactions has become a focal point. Overexpression of integrin α5β1, FAK, and LOX [5,8] provides a rationale for FAK inhibitors (defactinib) or targeting LOX/LOXL2, which is being explored as a translational strategy to impair invasive signaling in sarcomas [44]. Similarly, blockade of MFAP5—matricellular protein promoting angiogenesis and M2 macrophage recruitment—has shown anti-tumor activity in xenograft models [6,7].

Angiogenic mediators identified proteomically, including VEGFA, ANGPT2, and THBS1, align with the modest clinical benefit observed from anti-VEGF therapies such as bevacizumab or pazopanib. Proteomic-based stratification may improve the selection of patients most likely to respond to anti-angiogenic treatment [45].

#### 6.1.3. Immunomodulatory and Microenvironmental Targets

Proteomic signatures of immune suppression, such as elevated PD-L1, IDO1, ARG1, and CD163, suggest a rationale for immunotherapeutic combinations [7,33]. Preliminary data indicate that PD-1/PD-L1 blockade may yield partial responses in a subset of uLMS with high PD-L1 expression and interferon-γ signatures [33]. Integration of proteomic immune profiling with transcriptomic immune scores could guide personalized immunotherapy strategies [7].

### 6.2. Understanding Chemotherapy Resistance

#### 6.2.1. Proteomic Comparison of Chemo-Sensitive and Resistant Phenotypes

Standard chemotherapy for uLMS, primarily using doxorubicin, gemcitabine, and docetaxel, achieves limited durable benefit, with recurrence rates exceeding 70% [1]. Proteomics has elucidated multiple mechanisms underpinning this intrinsic or acquired resistance.

Comparative LC-MS/MS analyses between doxorubicin-sensitive and doxorubicin-resistant uLMS cell lines revealed overexpression of ATP-binding cassette (ABC) transporters—ABCB1 (P-gp) and ABCG2—facilitating drug efflux [21]. Resistance was further associated with upregulation of HSP27, HSP 90, and aldo-keto reductases (AKR1C1/3), both mitigating oxidative stress and apoptosis induced by anthracyclines [46]. Inhibition of HSP90 with ganetespib or onalespib resensitized resistant cells to doxorubicin in vitro [24].

Gemcitabine/docetaxel resistance has been linked to alterations in cytoskeletal and metabolic proteins. Label-free proteomics identified increased β-tubulin III (TUBB3) and stathmin 1 (STMN1) expression, leading to microtubule destabilization and impaired docetaxel binding [47]. Upregulation of glycolytic enzymes (ENO1, LDHA) and antioxidant proteins (PRDX1, SOD2) suggested metabolic reprogramming as a survival adaptation [48,49].

Moreover, phosphoproteomic profiling of resistant lines demonstrated hyperactivation of CHK1 and WEE1, sustaining DNA-damage tolerance [22,24]. Combination therapy with CHK1/WEE1 inhibitors restored doxorubicin sensitivity, indicating a potential synthetic-lethality approach.

#### 6.2.2. TME-Mediated Resistance

The TME contributes significantly to chemoresistance via paracrine signaling and matrix protection [7]. Proteomic analyses of conditioned media from uLMS-associated fibroblasts showed elevated MMP14, SERPINE1, and LOXL2, which enhance ECM stiffness and limit drug penetration [19,46]. Concurrently, macrophage-derived IL-10 and ARG1 activate STAT 3 signaling in tumor cells, promoting survival [7].

Secretome studies identified POSTN as a soluble mediator that enhances PI3K/AKT signaling and confers resistance to apoptosis [4,19,45]. Blocking POSTN or FAK signaling sensitized tumors to gemcitabine in xenograft models [19,22]. These findings highlight how proteomic dissection of TME interactions can uncover extracellular mechanisms of therapy failure.

#### 6.2.3. Post-Translational and Metabolic Adaptation

Proteomic and metabolomic integration reveals that chemotherapy triggers extensive post-translational remodeling. Increased phosphorylation of metabolic enzymes (PDHA1, PKM2) redirects pyruvate flux away from the TCA cycle, sustaining survival under drug stress [21,22]. Concurrent acetylation of HSP90 and α-tubulin modulates protein stability and microtubule dynamics, enhancing tolerance to cytotoxic agents [24]. These adaptive PTM networks suggest therapeutic opportunities using kinase inhibitors, HDAC inhibitors, or metabolic modulators to disrupt compensatory signaling [19,24].

### 6.3. Emerging Therapeutic Frameworks

#### 6.3.1. Proteomic-Guided Precision Medicine

Integrative multi-omics approaches are redefining therapeutic prioritization. Proteogenomic profiling links AURKA amplification and phospho-activation to aggressive behavior, supporting Aurora-kinase inhibition as a targeted strategy [21,24]. Similarly, proteomic confirmation of *TP53* pathway loss alongside mTORC1 activation rationalizes combined mTOR and DNA-damage-response blockade [24,30,39,50].

The ongoing emergence of DIA and single-cell proteomics enables fine-grained mapping of heterogeneity, which can inform individualized therapy [11,24]. Clinical implementation will require standardized workflows and cross-platform validation.

#### 6.3.2. Drug-Repurposing and Combination Strategies

Network-based drug-repurposing analyses using proteomic signatures have identified potential benefit from statins, metformin, and HDAC inhibitors, each modulating metabolic and epigenetic vulnerabilities [19,21,51]. Combination therapy targeting both kinase signaling (PI3K/mTOR) and metabolic reprogramming (glycolysis inhibitors) is currently being explored in preclinical sarcoma models [51]. While preclinical evidence for kinase and cell-cycle inhibitors (e.g., palbociclib, alisertib) is increasingly derived from uLMS-specific cell models, hypotheses regarding metabolic modulators such as statins and metformin largely rely on broader pan-sarcoma or general oncological mechanisms and warrant uLMS-specific validation.

Moreover, proteomics suggests synergy between immune checkpoint blockade and anti-angiogenic or mTOR inhibitors, leveraging complementary suppression of tumor metabolism and immune evasion [7,43].

## 7. Challenges, Limitations and Future Directions

Despite the significant advances in proteomic profiling of uLMS, several methodological, biological, and translational challenges limit the immediate clinical impact of these findings. This section highlights current obstacles, emerging strategies for multi-omics integration, and prospective pathways for clinical translation.

### 7.1. Current Limitations of uLMS Proteomics

#### 7.1.1. Sample Heterogeneity and Cohort Size

uLMS is a rare malignancy, comprising only 1 to 2% of uterine cancers, which inherently restricts available tissue for large scale studies [1,2,11]. Tumor heterogeneity further complicates proteomic analysis: variability in necrotic content, stromal admixture, and cellular composition can obscure biologically meaningful signals [5,6,23]. Microdissection and careful histopathologic annotation can mitigate these effects but increase labor and technical complexity [5].

Small cohort sizes in most studies limit statistical power and reproducibility, resulting in a high rate of candidate biomarker attrition during validation [1,6,11,19]. Multi-institutional collaborations and centralized tissue repositories are essential to overcome these constraints.

#### 7.1.2. Pre-Analytical and Technical Variability

Proteomic workflows are sensitive to pre-analytical factors such as tissue preservation, storage time, and lysis methods. While fresh-frozen tissue yields optimal protein recovery, FFPE samples are more widely available but present challenges due to formalin-induced crosslinking [11,24]. Differences in sample preparation, protein digestion, labeling, and MS instrumentation introduce variability that can obscure subtle biological differences [7,19,34]. Standardization of protocols and cross-platform benchmarking are critical to ensure reproducible results.

#### 7.1.3. Validation and Clinical Implementation Hurdles

Although numerous candidate diagnostic and prognostic markers have been identified—MFAP5, DPP6, TOP2A, AURKA, and ECM proteins—few have undergone rigorous, large-scale validation [6,11,19]. Assays such as IHC, ELISA, or targeted MRM-MS require standardization and verification in multi-center cohorts before routine clinical use. Additionally, regulatory approval pathways for proteomic biomarkers remain complex, further delaying translation [11,34].

### 7.2. Integrating Multi-Omics Data

Proteomic profiling alone provides a partial view of uLMS biology. Integration with genomics, transcriptomics, epigenomics, and metabolomics can enhance biological insight and identify actionable targets.

#### 7.2.1. Proteogenomics

Combining proteomic and genomic data allows direct assessment of how DNA alterations translate into functional protein changes. For instance, *TP53* or *RB1* mutations may be associated with altered phosphorylation of downstream targets or compensatory kinase pathway activation, revealing vulnerabilities not evident at the genomic level. Proteogenomic maps have already identified concordant dysregulation of PI3K/AKT/mTOR and cell-cycle proteins, guiding the development of targeted interventions [21,24,34].

#### 7.2.2. Integration with Metabolomics

Proteomic evidence of metabolic reprogramming, such as upregulation of glycolytic enzymes and altered TCA-cycle proteins, can be correlated with metabolite flux data to confirm pathway activation. Multi-omic integration strengthens mechanistic understanding and informs the rational design of metabolic inhibitors, such as LDHA or PDHA1 modulators, as adjunct therapies [17,22,39].

#### 7.2.3. Network and Systems Biology Approaches

Integrative network analysis combining proteomic, transcriptomic, and phosphoproteomic datasets can reveal central hubs and pathway crosstalk driving malignancy. Weighted co-expression networks, machine learning classifiers, and pathway enrichment analysis allow prioritization of biomarkers for diagnostic or therapeutic targeting, while reducing false-positive associations [6,8,19,24].

### 7.3. Clinical Translation

#### 7.3.1. Development of Reliable Assays

Translation of proteomic discoveries requires robust, reproducible assays suitable for clinical laboratories. Targeted MS (MRM or parallel reaction monitoring, PRM) and antibody-based platforms (IHC, ELISA, multiplexed immunoassays) are increasingly employed for candidate validation [11,24]. For example, MFAP5 and DPP6 have been validated by IHC, while TOP2A and AURKA are measured by targeted MS in research settings [6,11,19]. Standardized protocols for pre-analytical handling, instrument calibration and data normalization are essential to ensure reproducibility across institutions.

#### 7.3.2. Prospective Clinical Trials

The ultimate test of biomarker utility lies in prospective, multi-center trials that correlate protein expression with patient outcomes. Few such studies exist for uLMS, largely due to the rarity of the disease [1,11,19]. Embedding proteomic endpoints in ongoing trials—evaluating targeted therapies, chemotherapy regimens, or immunotherapy combinations—will provide real-world evidence for clinical implementation [21,24,39].

For therapeutic translation, companion diagnostics based on proteomic signatures could stratify patients for kinase inhibitors, metabolic modulators, or immunotherapies [21,24,52]. Integration with imaging and genomic biomarkers will likely be necessary for a truly precision-medicine approach.

#### 7.3.3. Challenges in Regulatory and Clinical Adoption

Proteomic biomarkers face regulatory hurdles, including demonstration of analytical validity, clinical validity, and clinical utility [11]. High-throughput discovery pipelines must be coupled with rigorous validation, reproducibility studies, and standardized reporting frameworks. Cost effectiveness, scalability, and accessibility also remain critical considerations for adoption in routine clinical care.

### 7.4. Future Directions

Future research in uLMS proteomics will benefit from expanded cohort studies and biobanking, with multi-institutional collaboration required to assemble sufficiently powered cohorts for both discovery and validation, including tissue and biofluid samples. Advances in single-cell and spatial proteomics will enable detailed mapping of intratumoral heterogeneity and TME interactions at cellular resolution, thereby refining biomarker discovery and revealing cell-type-specific vulnerabilities [7,8,51].

Continued progress in multi-omics integration—linking proteomic, genomic, transcriptomic, and metabolomic datasets—will generate more comprehensive molecular maps that support target prioritization and therapeutic decision-making [21,24,34]. Parallel to these efforts, the development of clinical-grade assays such as targeted proteomic panels, MRM-MS workflows, and multiplexed immunoassays is expected to transition the field from discovery-level experiments to clinically actionable diagnostics [11,24]. Finally, incorporating proteomic data into clinical trial design may help guide precision therapy, particularly in the context of kinase inhibitors, cell-cycle modulators, metabolic agents, and immunotherapy combinations [21,52].

## 8. Conclusions

uLMS remains a rare but highly aggressive malignancy with limited therapeutic options and poor prognosis [1,2,11]. Proteomic research has emerged as a critical tool for understanding the molecular underpinnings of this disease, offering complementary insights beyond genomic and histopathological analysis [6,7,12].

Through the application of advanced mass spectrometry techniques—including label-based quantitative methods (iTRAQ, TMT), label-free quantification, phosphoproteomics, and glycoproteomics—researchers have delineated key dysregulated pathways in uLMS. These include metabolic reprogramming consistent with the Warburg effect, ECM remodeling, cell-cycle deregulation, and post-translationally mediated signaling alterations [6,8,17,21]. Proteomic profiling has also highlighted the influential role of the TME, with immune suppression, stromal remodeling, and angiogenic signaling shaping tumor progression and therapy resistance [7].

Proteomic analyses have yielded a repertoire of candidate diagnostic and prognostic biomarkers, such as MFAP5, DPP6, TOP2A AURKA, and ECM-components (FN1, COL6A3, THBS2) [6,8,11,19,24]. These proteins not only differentiate uLMS from benign leiomyoma but also correlate with survival outcomes, disease recurrence, and chemotherapeutic response. Proteomic insights have further informed therapeutic targeting strategies, identifying vulnerabilities in kinase pathways, cell-cycle regulation, metabolic adaptation, and microenvironmental interactions [21,24,39].

However, substantial challenges remain. uLMS is a rare tumor with high intratumoral heterogeneity, complicating cohort assembly, reproducibility, and clinical validation [1,6,11,19]. Pre-analytical variability, standardization of proteomic workflows, and the translation of discovery-level findings into robust, clinically deployable assays remain critical hurdles [11,24].

The future of uLMS research lies in multi-omics integration, combining proteomic, genomic, transcriptomic, and metabolomic data to generate comprehensive, systems-level understanding of tumor biology [21,34]. Advancements in single-cell and spatial proteomics, coupled with classification models and network analysis, will enable high-resolution mapping of tumor heterogeneity, signaling networks, and microenvironmental interactions [7,8,24,51]. These integrated approaches are likely to facilitate personalized therapy, guiding patient-specific treatment decisions and combination strategies.

In conclusion, proteomics has transformed our understanding of uLMS biology, uncovering mechanistic insights, diagnostic markers, and therapeutic opportunities. With continued methodological refinement, large-scale validation, and integration into clinical trials, proteomic research holds the promise of significantly improving patient care through earlier diagnosis, more accurate prognostication, and precision-guided therapies.

## Figures and Tables

**Table 1:** Overview of quantitative proteomic strategies in uLMS ^1^ research.

Method	Description	Advantages
** iTRAQ/TMT isobaric labeling **	Chemical tagging allowing multiplexed sample quantification in one MS run and with small sample volume	High throughput; accurate relative quantification
** Label-free quantification **	Quantification of peptide abundance using intensity or spectral counts without labeling	Cost-effective and flexible; suitable for limited or rare samples
** Data-independent acquisition (DIA/SWATH-MS) **	Fragmentation of all ions within defined *m*/*z* windows for comprehensive, reproducible dataset	Enables robust comparisons across clinical cohorts; high reproducibility

^1^ uLMS: uterine leiomyosarcoma.

**Table 2:** Key protein biomarker candidates for uLMS **^1^** and current validation status.

Protein	Sample Type	Biomarker Type	Direction in uLMS ^1^	Validation Status * and Evidence	Study (Year)
** DPP6 **	Tissue	Diagnostic	Increased abundance	Orthogonal Validation: Transcriptomic correlation; network modeling of immune evasion	Ke et al. [5] (2022)
** MFAP5 **	Tissue	Diagnostic	Increased abundance	Orthogonal Validation: Transcriptomic correlation; in vivo xenograft assays	Ke et al. [5] (2022)
** TOP2A **	Tissue	Prognostic	Increased abundance	Orthogonal Validation/Clinical: Transcriptomic mapping; cell-cycle functional assays	Baiocchi et al. [28] (2016); Khamaiseh et al. [35] (2025)
** AURKA **	Tissue	Prognostic	Increased abundance	Orthogonal Validation: Gene amplification tracking; kinase activity assays	Savannah et al. [34] (2012); Guo et al. [6] (2024)
** CDK1 **	Tissue	Prognostic	Increased abundance	Discovery: Transcriptomic profiling; mitotic rate correlation	Khamaiseh et al. [35] (2025)
** Ki-67 **	Tissue	Prognostic	Increased abundance	Clinical/Orthogonal Validation: Immunohistochemical validation; meta-analyses	Travaglino et al. [29] (2021), Guo et al. [6] (2024)
** p16 (CDKN2A) **	Tissue	Diagnostic	Increased abundance	Orthogonal Validation: Genomic deletion/mutation correlation	Guo et al. [6] (2024)
** ECM proteins **	Tissue	Diagnostic	Altered (dysregulated ECM signature)	Discovery: In vitro cell-migration assays; ECM remodeling networks	Falcão et al. [25] (2025), Wang et al. [16] (2024)
** LDHA **	Serum/plasma	Characterization	Often elevated (but non-specific)	Clinical/large cohort: Metabolic flux assays (non-specific to uLMS)	Glorie et al. [36] (2019), Taliento et al. [37] (2025)
** Plasma proteins ** (e.g., albumin, immunoglobulins)	Serum/plasma	Characterization	Differentially abundant	Discovery: Proteomic profiling screening	Kopelman et al. [15] (2025)

^1^ uLMS: uterine leiomyosarcoma. * Validation Status: Discovery = identified by a proteomic or transcriptomic screen, not orthogonally validated; Orthogonal Validation = validated by IHC/Western/PRM/ELISA in the same or independent cohort; Clinical/large cohort = validated in multiple independent cohorts or assessed for diagnostic performance (AUC, sensitivity/specificity).

## Data Availability

No new data were created or analyzed in this study. Data sharing is not applicable to this article.

## Abbreviations

The following abbreviations are used in this manuscript:

2D-PAGE	Two-dimensional polyacrylamide gel electrophoresis
4EBP1	4E-binding protein 1
AKT	PKB (Protein Kinase B)
ALDOA	Aldolase A
ALDH1A1	Aldehyde dehydrogenase
ANGPT2	Angiopoietin-2
ABC	ATP-binding cassette
ARG1	Arginase 1
AURKA	Aurora kinase A
CA9	Carbonic anhydrase IX
CDK	Cyclin-dependent kinase
CCNB1	Cyclin B1
CHEK1	Checkpoint kinase 1
DIA	Data-independent acquisition
DPP6	Dipeptidyl-peptidase 6
ECM	Extracellular matrix
EGFR	Epidermal Growth Factor Receptor
ELISA	Enzyme-linked immunosorbent assay
ENG	Endoglin
ENO1	Enolase 1
EVs	Extracellular vesicles
FAK	Focal Adhesion Kinase
FN1	Fibronectin
GO	Gene ontology
HDAC	Histone deacetylase
HSP	Heat-shock proteins
HLA	Human Leukocyte Antigen
IDO1	Indoleamine 2,3-dioxygenase 1
IHC	Immunohistochemistry
IMS	Imaging mass spectrometry
iTRAQ	Isobaric tags for relative and absolute quantification
LDHA	Lactate dehydrogenase a
LCM	Laser-capture microdissection
LC-MS/MS	Liquid chromatography-tandem mass spectrometry
LFQ	Label-free quantification
LOX	Lysyl oxidase
MALDI	Matrix-assisted laser desorption/ionization
MFAP5	Microfibrillar-associated protein 5
MMP	Matrix metalloproteinase
MRM	Multiple-reaction monitoring
MS	Mass spectrometry
PDHA1	Pyruvate dehydrogenase E1 component a subunit
PI3K	Phosphoinositide 3-kinase
POSTN	Periostin
PRM	Parallel reaction monitoring
PTM	Post-translational modification
RPS6	Ribosomal protein S6
STAT3	Signal Transducer and Activator of Transcription 3
TCA	Tricarboxylic acid
TGF-β	Transforming growth factor-beta
THBS1	Thrombospondin-1
TME	Tumor microenvironment
TMT	Tandem mass tag
TOP2A	Topoisomerase II α
TPM1	Tropomyosin 1
uLMS	Uterine leiomyosarcoma
VEGF	Vascular endothelial growth factor
